# Flow Cytometry Reveals the Nature of Oncotic Cells

**DOI:** 10.3390/ijms20184379

**Published:** 2019-09-06

**Authors:** Anna Vossenkamper, Gary Warnes

**Affiliations:** 1 Centre for Immunobiology, The Blizard Institute, Barts and The London School of Medicine and Dentistry, Queen Mary London University, 4 Newark Street, London E1 2AT, UK; 2 Flow Cytometry Core Facility, The Blizard Institute, Barts and The London School of Medicine and Dentistry, Queen Mary London University, 4 Newark Street, London E1 2AT, UK

**Keywords:** accidental cell death, oncosis, DDR, parthanatos, flow cytometry

## Abstract

The term necrosis is commonly applied to cells that have died via a non-specific pathway or mechanism but strictly is the description of the degradation processes involved once the plasma membrane of the cell has lost integrity. The signalling pathways potentially involved in accidental cell death (ACD) or oncosis are under-studied. In this study, the flow cytometric analysis of the intracellular antigens involved in regulated cell death (RCD) revealed the phenotypic nature of cells undergoing oncosis or necrosis. Sodium azide induced oncosis but also classic apoptosis, which was blocked by zVAD (z-Vla-Ala-Asp(OMe)-fluoromethylketone). Oncotic cells were found to be viability^+ve^/caspase-3^–ve^/RIP3^+ve/–ve^ (Receptor-interacting serine/threonine protein kinase 3). These two cell populations also displayed a DNA damage response (DDR) phenotype pH2AX^+ve^/PARP^–ve^, cleaved PARP induced caspase independent apoptosis H2AX^–ve^/PARP^+ve^ and hyper-activation or parthanatos H2AX^+ve^/PARP^+ve^. Oncotic cells with phenotype cell viability^+ve^/RIP3^–ve^/caspase-3^–ve^ showed increased DDR and parthanatos. Necrostatin-1 down-regulated DDR in oncotic cells and increased sodium azide induced apoptosis. This flow cytometric approach to cell death research highlights the link between ACD and the RCD processes of programmed apoptosis and necrosis.

## 1. Introduction

The recent re-definition of cell death from Type I (programmed cell death by apoptosis), Type II (autophagic cell death), and Type III (programmed necrosis) to programmed cell death (PCD, homeostatic and embryonic), accidental cell death (ACD, oncosis), and regulated cell death (RCD), which includes apoptosis, necroptosis, autophagy, parthanatos or hyper-activation of Poly (ADP-ribose) polymerase (PARP) caused by an excessive DNA damage response (DDR, e.g., by pH2AX phospho H2AX histone) has been advantageous in understanding the complexity of cell death. RIP1- (Receptor-interacting serine/threonine protein kinase 1) dependent apoptosis or RIP1/RIP3/caspase-3 cells not being included highlights that the cell death nomenclature should be reviewed on a regular basis [1,2,3,4,5]. Programmed or regulated necrosis now includes necroptosis and parthanatos, amongst other forms of reported programmed cell death. However, necrosis is also the term commonly used to indicate the presence of dead cells that have lost plasma membrane integrity by any cell death pathway, but is strictly a reference to the degradation of the cell contents and plasma membrane after death [6,7,8,9,10]. The term oncosis or ACD is a better description of cell death induced by mechanical, chemical, and environmental factors that cause a rapid decrease in intracellular ATP leading to the deactivation of Na^+^ and K^+^-ATPase, resulting in an influx of Na^+^, Cl^–^, and Ca^2+^ ions. The cell then undergoes osmosis and swelling with a bursting of cell organelles and the plasma membrane [11]. Uncoupling protein 2 (UCP-2), located in the inner mitochondrial membrane where protons are pumped by UCP-2 into the mitochondrial matrix or the intermembrane space, which then regulates ATP and superoxide production is modestly up-regulated by oncosis, resulting in a rapid depolarization of the mitochondrial membrane, which has been measured by flow cytometry [12,13]. The signal pathways involved in oncosis are under studied, so little information is known about these mechanisms compared to the knowledge of pathways in RCD [1,2,6].

In contrast to oncosis, classic apoptosis is caspase-3 dependent and these cells form blebs on the surface of the plasma membrane with a gradual loss in mitochondrial membrane potential and hence a gradual lowering of intracellular ATP. The generation of reactive oxygen species (ROS) and loss of cytochrome *c* from the mitochondria to the cytoplasm results in activation of caspases and generation of apoptosomes and DNA fragmentation, accompanied with cell shrinkage and the formation of apoptotic bodies [1,2,8,14].

Until recently, necrotic or oncotic cells were measured via flow cytometry using the Annexin V assay in which such cells are gated as cell viability^+ve^/AnnexinV^–ve^ and numerous researchers have attempted to understand this dead cell population with varying success [13,15,16]. In our laboratory, mitochondrial and plasma membrane dysfunction were detected by the multiplexing of mitochondrial and plasma membrane probes into the Annexin V assay, leading to a better understanding of the biological processes occurring in this oncotic population [13,15,16]. The recent development of a polychromatic flow cytometric assay in this laboratory, which identifies most RCDs simultaneously and demonstrates pathways affected by use of pan-caspase and RIP1 protein blockers zVAD and necrostatin-1 (Nec-1), led us to re-investigate oncotic cell death for potential pathways by comparison with apoptosis. The markers measured by flow cytometry included a fixable cell viability marker, activated caspase-3 (apoptosis), up-regulated RIP3 (necroptosis, or resting when not), pH2AX (DDR), cleaved PARP (apoptosis), parthanatos, or hyper-activation of cleaved PARP (pH2AX/cleaved PARP; Table 1, Figure S1). Potential modulation of the oncotic response to sodium azide was further investigated by the use of zVAD and necrostatin-1 to evaluate if the oncotic signalling pathways can be modified by these inhibitors before the cell loses plasma membrane permeability and the cell undergoes oncosis [3,4,13,17,18,19]. This approach may indicate the nature of the oncotic cell phenotype and highlight potential mechanisms that can modify the oncotic cellular response and the ACD connection to RCD processes. This may increase the potential for the use of therapeutic drugs to target the ACD process in the treatment of cancer.

## 2. Results

### 2.1. Induction of Oncosis

**Figure 1:**
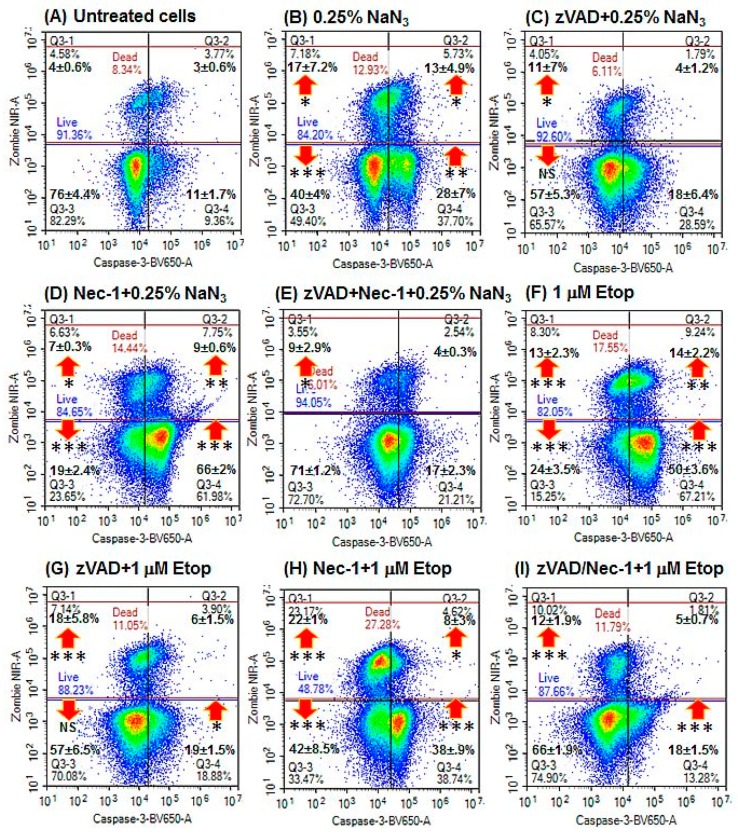
Cell death and caspase-3 activation assay. Cells were (**A**) untreated; (**B**) treated with 0.25% sodium azide (NaN_3_) for 24 h; (**C**) pre-treated with 20 µM zVAD for 2 h, then with 0.25% NaN_3_; (**D**) pre-treated with 60 μM necrostatin-1 (Nec-1) for 2 h, then with 0.25% NaN_3_; (**E**) pre-treated with 20 μM zVAD and 60 μM Nec-1 for 2 h, then with 0.25% NaN_3_; (**F**) treated with 1 μM Etoposide (Etop) for 24 h; (**G**) pre-treated with 20 μM zVAD for 2 h, then with 1 μM Etop; (**H**) pre-treated with 60 μM Nec-1 for 2 h, then with 1 μM Etop; and (**I**) pre-treated with 20 μM zVAD and 60 μM Nec-1 for 2 h, then with 1 μM Etop. *n* = 3, % Mean ± % SEM; Student’s *t*-test: NS (not significant), * *p* < 0.05, ** *p* < 0.01**, *** *p* < 0.001; arrows indicate change compared with untreated cells.

**Figure 2:**
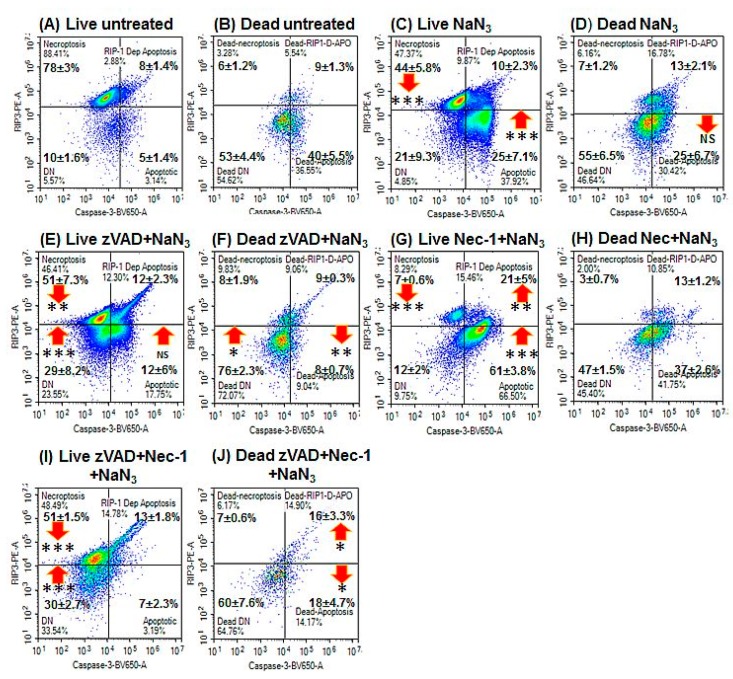
RIP3 and caspase-3 activation analysis of oncosis. After gating on live and dead cells from a Zombie NIR vs. caspase3-BV650 dot-plot (**A**) untreated live and (**B**) dead Jurkat cells were analysed on a RIP3-PE vs. caspase-3-BV650 dot-plot with resting phenotype indicated by RIP3^+ve^/caspase-3^–ve^, apoptosis by RIP3^–ve^/caspase-3^+ve^, RIP1-dependent apoptosis RIP3^+ve^/caspase-3^+ve^, and double negative RIP3^–ve^/caspase-3^–ve^. Live and dead cells treated with (**C,D**) 0.25% NaN_3_ for 24 h; (**E**,**F**) pre-treated with 20 μM zVAD for 2 h, then treated with 0.25% NaN_3_; (**G**,**H**)pre-treated with 60 μM Nec-1 for 2 h, then treated with 0.25% NaN_3_; and (**I**,**J**) pre-treated with 20 μM zVAD and 60 μM Nec-1 for 2 h, then treated with 0.25% NaN_3_, respectively. *n* = 3, % Mean ± % SEM, Student’s *t*–test: NS (not significant), * *p* < 0.05, ** *p* < 0.01**, *** *p* < 0.001; arrows indicate change compared with untreated cells.

NaN_3_ induced early apoptosis (28%, Figure 1B, lower right quadrant) and lower levels of late apoptotic (13%, Figure 1B, upper right quadrant) and oncotic cells (17%, Figure 1B, upper left quadrant) compared with untreated cells after 24 h (Figure 1A,B, see Materials and Methods section for details of cell phenotype and gating strategy, Table 1, Figure S1). A lower incidence of live resting cells was observed (RIP3^+ve^/caspase-3^–ve^, 44%, Figure 2C, upper left quadrant) but with more early apoptosis after treatment (RIP3^–ve^/caspase-3^+ve^, 25%, Figure 2A,C, lower right quadrant). Dead cells arising from NaN_3_ treatment showed less late apoptosis (25%, Figure 2D, lower right quadrant) than untreated cells (Figure 2B).

The oncotic cells resulting from NaN_3_ treatment were mainly double negative (55%) for RIP3 and caspase-3 expression (dead resting oncotic cells, <10% caspase-3^–ve^/RIP3^+ve^, Figure 2D).

**Figure 3:**
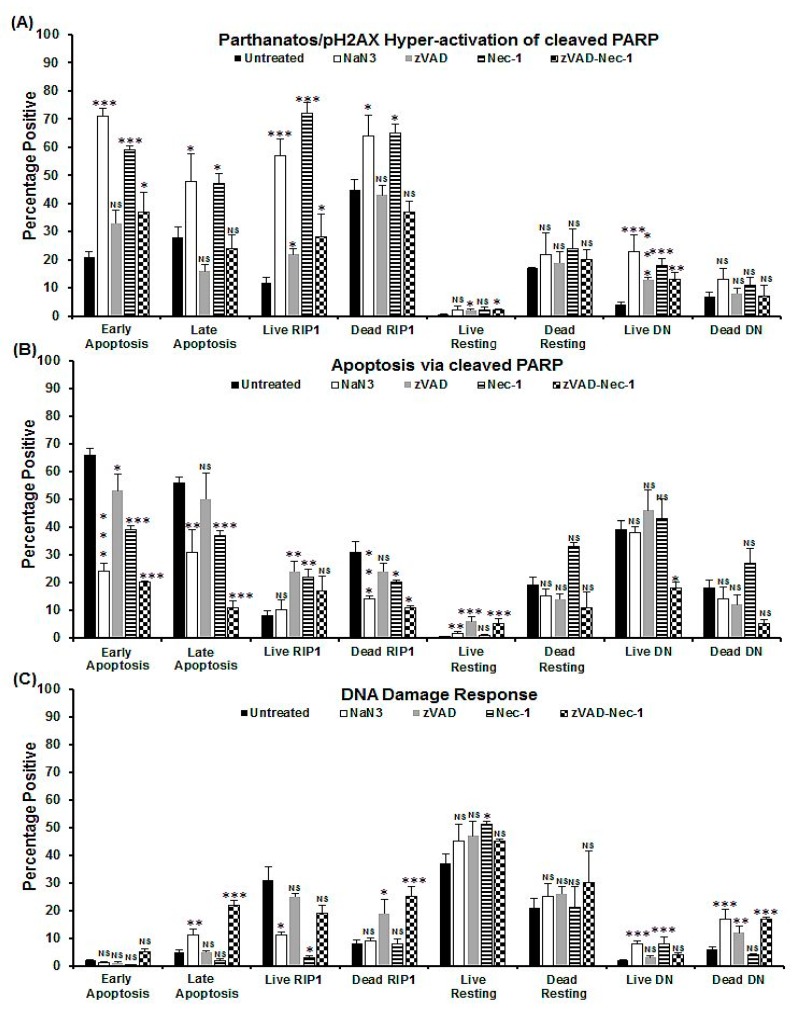
Parthanatos/hyper-activation of cleaved PARP, apoptosis via cleaved PARP, and DDR analysis of oncosis. Untreated Jurkat cells, treated with 0.25% NaN_3_ for 24 h, or pre-treated with zVAD (20 μM) and/or Nec-1 (60 μM) for 2 h, then incubated with 0.25% NaN_3_. Gating live and dead cells from a Zombie NIR vs. caspase-3-BV650 plot then both were analysed on a RIP3-PE vs. caspase-3-BV650 plot. Next, early and late apoptotic, necroptotic/resting, RIP1-dependent apoptotic, and double negative (DN) populations were analysed for pH2AX and cleaved PARP (Figures S2, S3). The incidence of (**A**) parthanatos/hyper-activation of cleaved PARP, (**B**) apoptosis via cleaved PARP, and (**C**) DDR were determined for all populations listed above. *n* = 3, % Mean, error bars % SEM, Student’s *t*-test; NS (not significant), * *p* < 0.05, ** *p* < 0.01**, *** *p* < 0.001 compared with untreated cells.

After NaN_3_ treatment, the two live and dead apoptotic populations showed increased levels of pH2AX hyper-activation of cleaved PARP and a lower degree of apoptosis via cleaved PARP and DDR than untreated cells (Figure 3A–C, Figure S1A,B,E,F,I,J,M,N). Whereas late apoptotic cells showed increased DDR (Figure 3C, Figure S2B,E,M,J). The dead resting oncotic cells (Zombie^+ve^/caspase-3^–ve^/RIP3^+ve^) were, 31% negative for both H2AX and PARP, whereas the dead oncotic DN (Zombie^+ve^/caspase-3^–ve^/RIP3^–ve^) cells were 57% negative for both markers (Figure 3A–C, Figure S2O,P). The live and dead DN populations showed increased levels of parthanatos and DDR (Figure 3A–C, Figure S2D,H,L,P).

### 2.2. Induction of Apoptosis

**Figure 4:**
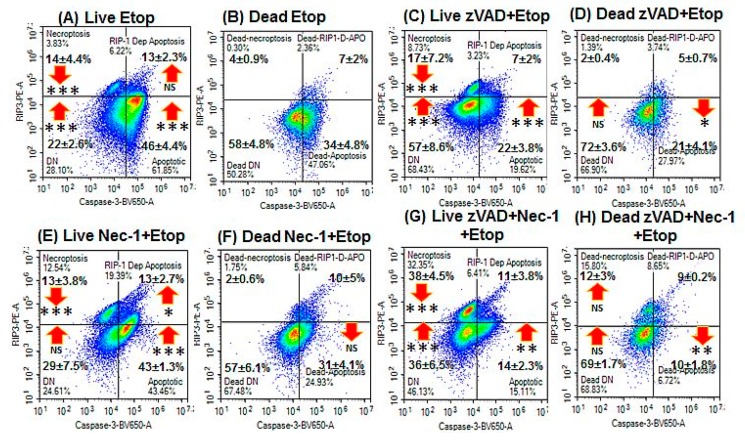
RIP3 and caspase-3 activation analysis of apoptosis. Gating on live and dead cells from a Zombie NIR vs. caspase-3-BV650 plot followed by analysis on a RIP3-PE vs. caspase-3-BV650 plot with resting phenotype indicated by RIP3^+ve^/caspase-3^–ve^, apoptosis by RIP3^–ve^/caspase-3^+ve^, RIP1-dependent apoptosis by RIP3^+ve^/caspase-3^+ve^ and double negative by RIP3^–ve^/caspase-3^–ve^. (**A,B**) Treated with 1 μM Etop for 24 h; (**C**,**D**), pre-treated with 20 μM zVAD for 2 h, then treated with 1 μM Etop; (**E**,**F**) pre-treated with 60 μM Nec-1 for 2 h, then treated with 1 μM Etop; and (**G**,**H**) pre-treated with 20 μM zVAD and 60 μM Nec-1 for 2 h, then treated with 1 μM Etop. *N* = 3, % Mean ± % SEM, Student’s *t*–test: NS (not significant), * *p* < 0.05, ** *p* < 0.01**, *** *p* < 0.001; arrows indicate change compared with untreated cells.

Induction of apoptosis with Etop showed an increase in early and late apoptosis as well as oncotic cells compared with untreated cells (Figure 1A,F). Live and early apoptotic cells showed increased levels of both types of apoptosis and the DN cells, whereas dead cells showed no such change (Figure 2A,B and Figure 4A,B).

**Figure 5:**
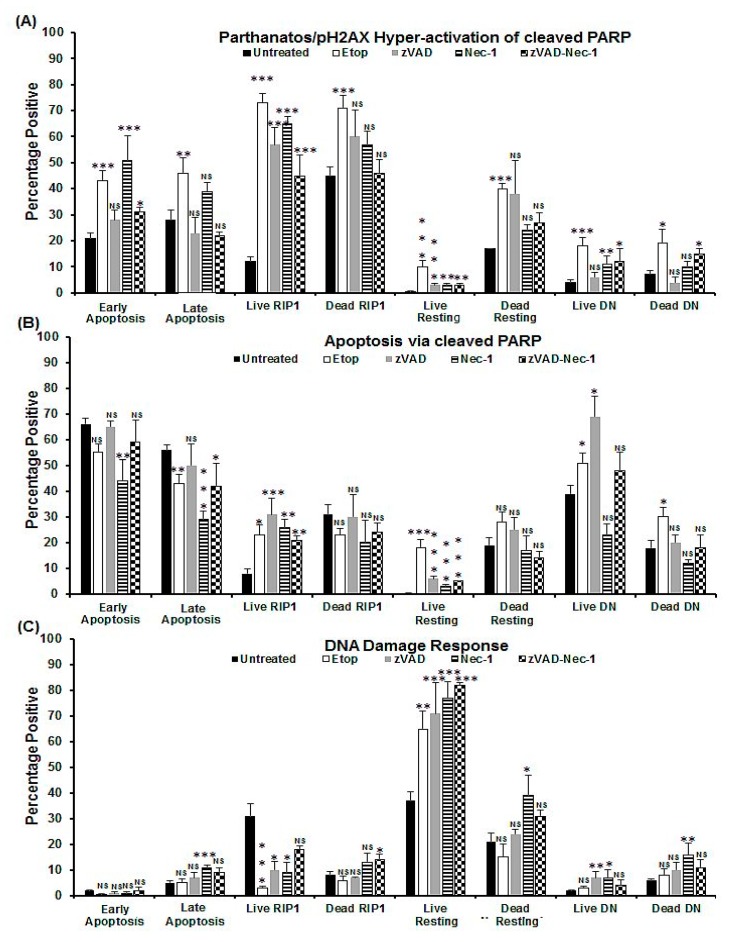
Parthanatos/hyper-activation of cleaved PARP, apoptosis via cleaved PARP, and DDR analysis of apoptosis. Untreated Jurkat, treated with 1 μM Etop or pre-treated with zVAD (20 μM) and/or Nec-1 (60 μM) for 2 h, then incubated with 1 μM Etop for 24 h. Gating on live and dead cells from a Zombie NIR vs. caspase-3-BV650 plot then both were analysed on a RIP3-PE vs. caspase-3-BV650 plot. Early and late apoptotic, necroptotic/resting, RIP1-dependent apoptotic, and double negative (DN) populations were analysed for pH2AX and cleaved PARP (Figures S2,S4 for detailed information). The incidence of (**A**) parthanatos/hyper-activation of cleaved PARP, (**B**) apoptosis via cleaved PARP, and (**C**) DDR were determined for all populations listed above. Mean, error bars % SEM, Student’s *t*-test: NS (not significant), * *p* < 0.05, ** *p* < 0.01**, *** *p* < 0.001 compared with untreated cells.

Early and late apoptotic cells after Etop treatment showed increased pH2AX hyper-activation of cleaved PARP with a decrease in apoptosis via cleaved PARP compared with untreated cells (Figure 5A,B and Figure S2Q,U). Live RIP1-dependent cells, however, showed increased pH2AX hyper-activation of cleaved PARP and caspase-3-dependent apoptosis via cleaved PARP with decreased DDR (Figure 5A–C and Figure S2R). However, live and dead oncotic resting and DN phenotypes also showed increased parthanatos and caspase-3-independent apoptosis via cleaved PARP with no increase in DDR, except for an increase observed in the live resting population (Figure 5A–C and Figure S2S,T,V,W,X).

### 2.3. Blockade of Caspases

Pre-treatment with zVAD to block the activation of caspases by NaN_3_ and Etop resulted in lower levels of early apoptosis (<20%) and late apoptosis (<10%) with no change in the incidence of oncotic cells (Figure 1C,G, Figure 2E,F and Figure 4C,D). The proportion of live DN (RIP3^–ve^/caspase-3^–ve^) and dead oncotic cells (RIP3^–ve^/caspase-3^–ve^) increased after zVAD blockade of both drugs (Figure 2E,F and Figure 4C,D).

After zVAD caspase blockade of NaN_3_ and Etop treatments, all live populations showed increased H2AX hyper-activation of cleaved PARP or parthanatos compared with untreated cells but decreased compared to drugs alone (Figure 3A, Figure 5A and Figure S3A–D, S4A–D). The live RIP1-dependent apoptotic, resting, and DN cells from both treatments also showed increased levels of apoptosis via cleaved PARP compared with untreated cells (early apoptosis showed a decrease, Figure 3B, Figure 5B and Figure S3A–D, S4A–D). The live DN population after zVAD/NaN_3_ or Etop treatment showed a decrease or increase of DDR compared with drugs alone, respectively (Figure 3C, Figure 5C and Figure S3D, S4D). Live RIP1-dependent apoptotic and resting phenotypes, after both treatments with zVAD, showed increased DDR compared with drugs alone (Figure 3C, Figure 5C and Figure S3B,C, S4B,C). Dead cells from zVAD blockade of NaN_3_/Etop treatments returned pH2AX and cleaved PARP expression to that of untreated dead cells (Figure 3, Figure 5 and Figure S3E–H, S4E–H). Except after NaN_3_/zVAD treatment, an increase in DDR was observed in the dead RIP1-dependent apoptotic and oncotic DN phenotypes (Figure 5 and Figure S3F,H).

### 2.4. Blockade with Necrostatin-1

Blockade of NaN_3_ with Nec-1 resulted in very high levels of early apoptosis compared with NaN_3_, but was lower with Etop treatment (Figure 1D,H). Cell death was lower with Nec-1/NaN_3_ but not changed with Etop treatment (Figure 1D,H). The live cells showed a higher incidence of early and RIP1-dependent apoptosis compared with NaN_3_ treatment, with no change observed with Etop or in the incidence of dead cells (Figure 2A–D,G,H and Figure 4A,E,F).

pHA2X hyper-activation of cleaved PARP in live and dead cells after Nec-1 showed increased values similar to that observed with only drugs, except for the lower levels in both the DN populations and live resting cells after Nec-1/Etop treatment (Figure 3A, Figure 5A and Figure S3I–P, S4I–P). Apoptosis via cleaved PARP was increased in live and dead cells after Nec-1 blockade of NaN_3_ treatment and decreased with Nec-1/Etop treatment compared with drugs alone, except for a decrease in live resting cells (Nec-1/NaN_3_) and no change in RIP1-dependent apoptosis after Nec-1/Etop treatment (Figure 3B, Figure 5B and Figure S3I–P, S4I–P). Very low levels of DDR were observed in early and live RIP1-dependent apoptotic cells, but increased in live resting and DN cells (Figure 3C, Figure 5C and Figure S3I–L, S4I–L). Dead cell phenotypes after Nec-1 blockade of NaN_3_ showed no increase in DDR compared with untreated cells, but was increased with Nec-1/Etop treatment (Figure 3C, Figure 5C and Figure S3, S4M–P).

### 2.5. Blockade with zVAD and Necrostatin-1

Pre-treatment with zVAD and Nec-1 to block the activation of caspases and RIP proteins resulted in lower levels of early and late apoptosis (<20%), although oncosis (caspase-3^–ve^/Zombie^+ve^) was still maintained (Figure 1E,I, Figure 2I,J and Figure 4G,H). Blocked NaN_3_ treated cells had a higher level of live DN cells (and Etop), whereas dead cells showed higher levels of RIP1-dependent apoptosis, which indicated that zVAD did not block caspases in the RIP1-dependent apoptotic pathway in the presence of Nec-1 (Figure 2I,J and Figure 4G,H).

All cell phenotypes after dual blockade showed the same reduced levels of pH2AX hyper-activation of cleaved PARP as that observed after zVAD blockade (no change in Etop DN cells, Figure 3A, Figure 5A and Figure S3Q–X, S4Q–X). Apoptosis via cleaved PARP was lower in the early apoptotic and DN cells after dual blockade of NaN_3_ and increased in live resting and RIP1-dependent apoptotic cells (Figure 3B and Figure S3Q,T). In contrast, all live cell (dual-blocked Etop) populations showed no change in apoptosis via cleaved PARP compared with drug treatment, except for the lower levels observed in live resting cells (Figure 5B and Figure S4Q,T). All dead cell populations after dual blockade of both treatments showed lower levels of apoptosis via cleaved PARP compared to any treatment protocol, except no change was observed in Etop-induced late and RIP1-dependent apoptotic cells (Figure 3B, Figure 5B and Figure S3U–X, S4U–X). Lastly, the DDR levels after both treatment protocols showed that all cell populations had higher levels than untreated cells (Figure 3C and Figure 5C), except for the lower levels found in the live RIP1-dependent apoptotic cells compared with both treatments (Figure 3C and Figure 5C).

## 3. Discussion

**Figure 6:**
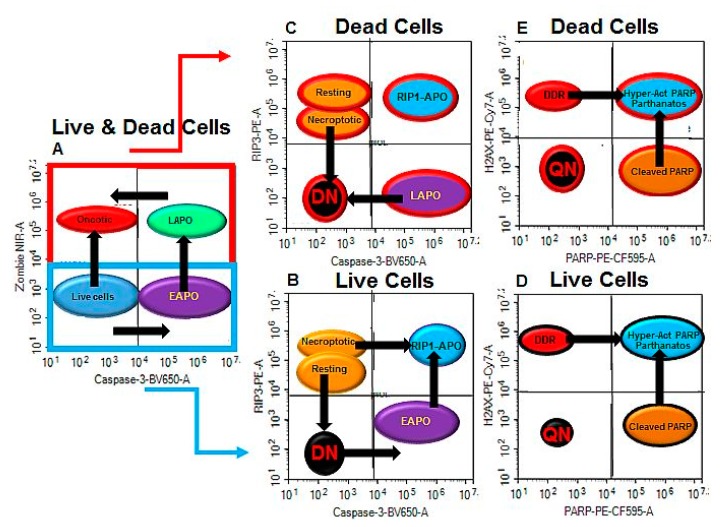
ACD and RCD pathways. (**A**) Live cells can undergo either early apoptosis (EAPO) or oncosis after drug treatment, with early apoptotic cells moving to late apoptotic (LAPO), then later with cell degradation, to the oncotic or necrotic phenotype. (**B**) Live cells may express RIP3^+ve^/caspase-3^–ve^ when resting, or be RIP3^high+ve^/caspase-3^–ve^ when undergoing necroptosis, or be double negative (DN). EAPO cells lose RIP3 or, if retained, undergo RIP1-dependent apoptosis (RIP1-APO). EAPO cells can also become RIP1^+ve^. (**C**) Loss of plasma membrane integrity or cell death results in cell phenotypes mirrored in (**B**), with degradation of cells resulting in the DN population. (**B**,**C**) Live and dead cell phenotypes can also express pH2AX (DDR) or cleaved PARP (apoptosis), both of which can ultimately express both proteins, (**D**,**E**) resulting in pH2AX hyper-activation of cleaved PARP in the presence of active caspase-3 or parthanatos in the absence of caspase-3. Arrows indicate movement of cell populations.

The use of oncosis and apoptosis-inducing drugs NaN_3_ and Etop, together with caspase and RIP protein blockers, zVAD and Nec-1, has allowed the tracking of the cell death processes involved in ACD and apoptosis a form of RCD using flow cytometry (Figure 6) [17,18,19]. Induction of oncosis or apoptosis resulted in measurable oncosis (Zombie^+ve^/caspase-3^–ve^, commonly termed necrotic) but also early and late apoptosis, which was reduced by zVAD blockade without affecting the degree of oncosis induced by both drugs. Blockade of both drugs with Nec-1 resulted in increased NaN_3_-induced early apoptosis while increasing Etop-induced oncosis. Induction of ACD and early apoptosis (by NaN_3_) showed that live resting Jurkat cells move the RIP3^+ve^/caspase-3^–ve^/Zombie^–ve^ phenotype to the early apoptotic phenotype of RIP3^–ve^/caspase-3^+ve^/Zombie^–ve^, then later to the RIP1-dependent phenotype RIP3^+ve^/caspase-3^+ve^/Zombie^–ve^ (Figure 6B), even in the presence of RIP1 inhibitor, Nec-1. This effect has been previously reported [17,19], where it was shown that although Nec-1 inhibited necroptosis by abrogation of the up-regulation of RIP3 (RIP3^+ve^/caspase-3^–ve^/Zombie^–ve^), it did not inhibit cells from undergoing apparent RIP1-dependent apoptosis. The limitation of the current assay is highlighted by the use of RIP3 and caspase-3 antibodies to indirectly identify RIP1-dependent apoptosis due to the lack availability of a fluorescenated RIP1 antibody [17,19]. The interactions of RIP1, RIP3, TRADD (TNFR1-associated death domain), FADD (Fas associated via death domain), and caspase-8 in apoptotic Complex IIa and IIb pathways are not completely understood, so another explanation of the apparent presence (indirectly via RIP3) of RIP1-dependent apoptosis in the presence of Nec-1 is required, which may be elucidated by the use of a fluorescenated RIP1 antibody [5,20,21].

Induction of apoptosis by Etop showed that a high proportion of live resting cells become DN (losing their RIP3), as well as another population expressing caspase-3, possibly indicating that the cells first lose RIP3, become DN, and then express caspase-3 (Figure 6B), but also that early apoptotic cells can also later express RIP3 (Figure 6B) [5,17,19,20,21]. The route to RIP1-dependent apoptosis may be that these resting cells, rather than lose their RIP3, also start to express caspase-3 (Figure 6B).

Once the cells lose plasma membrane integrity and become Zombie^+ve^, the cells presumably maintain the late apoptotic phenotype RIP3^–ve^/caspase-3^+ve^/Zombie^+ve^ before further degradation resulting in the cells becoming DN (Figure 6C). Oncotic cells (caspase-3^–ve^/Zombie^+ve^) induced by NaN_3_/Etop, however, can also be divided into those with RIP3^+ve^/caspase-3^–ve^/Zombie^+ve^ or the DN phenotype RIP3^–ve^/caspase-3^–ve^/Zombie^+ve^ (Figure 6C).

The expression of pH2AX and cleaved PARP in the four identified phenotypes in live and dead cells are resting, early or late apoptotic, RIP1-dependent apoptosis, and DN, the incidence of which is modified by the action of the two drugs used in this study and can be further manipulated by blockade of ACD and RCD processes by zVAD and Nec-1 (Figure 6D,E) [22,23,24,25]. In the first instance live resting cells, the main phenotype of untreated Jurkat cells express RIP3 with a high degree of DDR (37%), whereas resting DN cells showed little DDR (2%) but a high degree of cleaved PARP (39%) in the absence of caspase-3 [25]. Induction of ACD resulted in enhanced levels of pHA2X hyper-activation of cleaved PARP in all live cell phenotypes with consequent reduced levels of DDR in live RIP1-dependent apoptotic cells, but with increased levels in the live DN phenotype (Figure 6D).

Once the cells undergo death, all phenotypes still showed increased pHA2X hyper-activation of cleaved PARP above the levels observed with dead untreated Jurkat cells (Figure 6E), whereas late apoptotic and dead oncotic DN cells showed increased DDR with no change in dead resting cells. Similar results were observed when cells undergo Etop-induced apoptosis, except no increase in DDR was observed in the dead oncotic or resting cells. So, NaN_3_-derived oncotic cells can express more DDR than Etop-induced oncotic cells, which expressed higher levels of cleaved PARP (in the absence of caspase-3) and parthanatos/pHA2X hyper-activation of cleaved PARP, again in the absence of caspase-3 (Figure 6E).

The main effect of blockade with zVAD was an increased incidence of live and dead DN cells with both treatments, which displayed no change in DDR, increased cleaved PARP and reductions in pHA2X hyper-activation of cleaved PARP, as was the case with most cell phenotypes (Figure 6D, E). This was with the notable exception of no change in levels of cleaved PARP in dead oncotic resting and DN cells compared to NaN_3_ treatment.

In contrast, blockade of NaN_3_ with Nec-1 resulted in no change in the incidence of dead DN and fewer live DN cells (as untreated cells), whereas Etop-induced levels were similar to that observed by drug alone. However, all blocked NaN_3_ cell phenotypes showed increased cleaved PARP and pH2AX activation of cleaved PARP with reductions in DDR; this observation was especially noteworthy in the dead oncotic resting and DN cells, whereas the opposite was observed in these populations after Nec-1 blockade of Etop.

Dual blockade of both treatments again showed a high incidence of DN cells with reduced levels of pHA2X hyper-activation of cleaved PARP and cleaved PARP expression in the various populations of cells, whereas DDR was generally increased after Nec-1 blockade of both drugs in most populations of cells. So, the blockade of apoptotic and/or necroptotic pathways when the cells are undergoing ACD and RCD processes radically increased the incidence of oncotic cells, especially in the case of blockade of apoptosis alone and necroptosis, which resulted in increased levels of DDR, whereas Nec-1 blockade reduced ACD-related DDR but not in the case of RCD.

For decades, oncotic cells have been an undetermined population of dead cells that have been overlooked due to the difficult in their characterisation rather than being a point of interest. They have been detected by use of the Annexin V assay and classed as cell viability^+ve^/Annexin V^–ve^ [13,15,16]. Using an active caspase-3 and RIP3 antibodies in tandem with a fixable live/dead dye, we showed that these oncotic cells have two main phenotypes, both of which are cell viability^+ve^/caspase-3^–ve^/RIP3^+/–ve^, which can be further divided into DDR, hyper-activation of PARP or parthanatos, apoptosis via PARP or cells undergoing programmed necrosis, as well as double negative oncotic cells. All of them are expressed to different degrees compared with cells derived from untreated cultures. This perhaps reflects their different origins, with differences in expression of these markers when the cells are derived from an ACD or RCD induction process. These differences in origin are further highlighted by their differing oncotic responses to blockade by zVAD and Nec-1 or both.

## 4. Materials and Methods

### 4.1. Induction of Oncosis and Apoptosis

Jurkat cells (human acute T cell leukaemia cell line, ECACC, Salisbury, UK) were grown in RPMI (Roswell Park Memorial Institute 1640 Medium) 1640 with 10% FBS (Fetal Bovine Serum, Invitrogen, Paisley, UK) at 37 °C and 5% CO_2_, either untreated or treated with 0.25% sodium azide or 1 μM etoposide (NaN_3_, Etop, Sigma, Poole, UK) for 24 h. Cells were pre-treated with pan-caspase blocker zVAD (20 μM, Enzo Life Sciences, Exeter, UK) and/or necroptosis blocker necrostatin-1 (Nec-1, 60 μM Cambridge Bioscience, Cambridge, UK) for 2 h before induction of oncosis with 0.25% sodium azide or apoptosis with 1 μM Etop for 24 h.

### 4.2. Flow Cytometry Assay

Harvested cells were labelled with fixable live dead stain, Zombie near infra red (NIR; Biolegend, San Diego, CA, USA) at room temperature (RT) for 15 min. Washed cell were fixed in Solution A (CalTag, Little Balmer, UK) then 0.25% Triton X-100 (Sigma, Poole, UK) for 15 min each at RT. Jurkat cells (1 × 10^6^) were incubated for 20 min at RT with anti-RIP3-PE (phycoerythrin clone B-2, Cat. No. sc-374639, Santa Cruz, Dallas, Tx, USA), cleaved PARP-PE-CF-595 (clone F21-852, Becton Dickinson, San Jose, CA, USA), H2A.X-Phospho (ser139)-PE-Cy7 (clone 2F3, Biolegend, San Diego, CA, USA) and anti- active caspase-3-BV650 (clone C92-605, Becton Dickinson, San Jose, CA, USA) for 20 min at RT. Washed cells were resuspended in 400 μL PBS (Phosphate Buffered Saline) and analysed on a ACEA Bioscience Novocyte 3000 flow cytometer (100,000 events, San Diego, CA, USA). Zombie NIR was excited by a 633 nm laser and collected with a 780/60 nm detector. Caspase-3-BV650 was excited by a 405 nm laser and collected at 675/30 nm. RIP3-PE, cleaved PARP-PE-CF-595, and pH2AX-PE-Cy7 were excited by a 488 nm laser and collected at 572/28, 615/20, and 780/60 nm, respectively. Single colour controls were used to determine the colour compensation using the pre-set voltages on the instrument using Novo Express software (ver 1.2.5, ACEA Biosciences, San Diego, CA, USA). Cells were gated on FSC (Forward Scatter) vs. SSC (Side Scatter) with single cells being gated on a FSC-A (Area) vs. FSC-H (Height) plot. Cells were then gated on a plot of caspase-3-BV650 vs. Zombie NIR, with a quadrant placed marking off live cells in the double negative quadrant (Figure S1A, lower left quadrant), with caspase-3-BV650^+ve^/Zombie NIR^–ve^ (Figure S1A, lower right quadrant) indicating early apoptotic cells (EAPO), and lastly with caspase-3-BV650^+ve^/Zombie NIR^+ve^ and caspase-3-BV650^–ve^/Zombie NIR^+ve^ (Figure S1A, upper quadrants) indicating dead late apoptotic (LAPO) (Figure S1A, upper right quadrant) and oncotic cells (Figure S1A, upper left quadrant). Further labelling with RIP3 allowed identification within the live resting and dead oncotic populations of RIP3^+ve^/caspase-3^–ve^ or necroptotic cells when RIP3 is up-regulated, early or late apoptotic (RIP3^–ve^/caspase-3^+ve^), RIP1-dependent apoptosis (RIP3^+ve^/caspase-3^+ve^, RIP1APO), and live double negative (DN) or dead oncotic DN cells (Figure S1B,D). Further gating on each of these eight populations for pH2AX and cleaved PARP allowed the identification of DDR (H2AX^+ve^/PARP^–ve^), pH2AX hyper-activation of cleaved PARP or parthanatos (H2AX^+ve^/PARP^+ve^), apoptotic cell death via cleaved PARP (H2AX^–ve^/PARP^+ve^), and DN cells (H2AX^–ve^/PARP^–ve^) (Figure S1C,E) [19]. In particular, dead resting oncotic cells (Zombie^+ve^/caspase-3^–ve^/RIP3^+ve^) and dead oncotic DN cells (Zombie^+ve^/caspase-3^–ve^/RIP3^–ve^) were gated for pH2AX and cleaved PARP, revealing the phenotypic nature of these two types of oncotic cells (Figure S1F,G).

### 4.3. Statistics

For all experiments, *n* = 3 and data are reported as mean ± SEM for percentage positive. Student’s *t-*tests were performed in GraphPad software Inc. (San Diego, CA, USA) with *p* ≥ 0.05 considered not significant (NS). * denotes *p* ≤ 0.05, ** denotes *p* ≤ 0.01, and *** denotes *p* ≤ 0.001 when treated cells were compared to untreated cells.

## Figures and Tables

**Table 1:** Cell description and phenotypes; Figure S1 provides a diagrammatical representation.

Cell Population	Phenotypic Markers
Live resting (or necroptotic)	Caspase-3^–ve^/Zombie NIR^–ve^/RIP3^+ve^
Live double negative (DN)	Caspase-3^–ve^/Zombie NIR^–ve^/RIP3^–ve^
Early apoptosis (EAPO)	Caspase-3^+ve^/Zombie NIR^–ve^/RIP3^–ve^
Live RIP1-dependent apoptosis (RIP1-APO)	Caspase-3^+ve^/Zombie NIR^–ve^/RIP3^+ve^
Late apoptosis (LAPO)	Caspase-3^+ve^/Zombie NIR^+ve^/RIP3^–ve^
Dead/necrotic/oncotic	Caspase-3^–ve^/Zombie NIR^+ve^
Dead resting (or necroptotic)	Caspase-3^–ve^/Zombie NIR^+ve^/RIP3^+ve^
Dead double negative (DN)	Caspase-3^–ve^/Zombie NIR^+ve^/RIP3^–ve^
Dead RIP1-dependent apoptosis (RIP1-APO)	Caspase-3^+ve^/Zombie NIR^+ve^/RIP3^+ve^
DNA damage response (DDR)	pH2AX^+ve^/Cleaved PARP^–ve^
Hyper-activation of cleaved PARP/parthanatos	pH2AX^+ve^/Cleaved PARP^+ve^
Cleaved PARP	pH2AX^–ve^/Cleaved PARP^+ve^

## Supplementary Materials

Supplementary materials can be found at https://www.mdpi.com/1422-0067/20/18/4379/s1.
