# Supplementary material for: Flow Cytometry Reveals the Nature of Oncotic Cells
Source: Int J Mol Sci. 2019 Sep 6;20(18):4379. doi: 10.3390/ijms20184379 (PMC6769836; doi:10.3390/ijms20184379)
Supplement: Supplementary file 1 [file ijms-20-04379-s001.pdf]

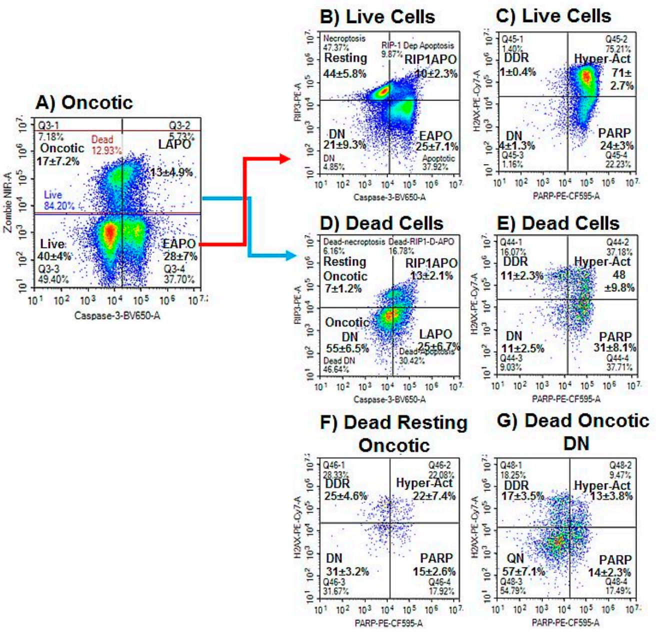

Figure 1. Immunophenotyping gating strategy of Regulated Cell Death. Live (including early apoptotic) and dead cells including late apoptotic and necrotic were gated from a Zombie NIR vs. Caspase-3-BV650 dot-plot (A). Live and dead resting were defined as Caspase-3^{-ve}/RIP3^{+ve}, early or late apoptotic (Caspase-3^{+ve}/RIP3^{-ve}), RIP1-dependent apoptotic (RIP1-APO, Caspase-3^{+ve}/RIP3^{+ve}) and Double Negative (DN, Caspase-3^{-ve}/RIP3^{-ve}) (B, D). Then each of these live and dead cell populations were then gated on a H2AX vs. cleaved PARP dot-plots to show the incidence of DDR (H2AX^{+ve}/PARP^{-ve}), hyper-activation of cleaved PARP (pH2AX^{+ve}/PARP^{+ve}), apoptosis via cleaved PARP (H2AX^{-ve}/PARP^{+ve}) and DN (pH2AX^{-ve}/PARP^{-ve}), (D, E). Dead resting oncotic (F, Zombie^{+ve}/Caspase-3^{-ve}/RIP3^{+ve}) and dead oncotic DN cells (G, Zombie^{+ve}/Caspase-3^{-ve}/RIP3^{-ve}) were then analysed on pH2AX vs. cleaved PARP dot-plots (QN, quadruple negative).

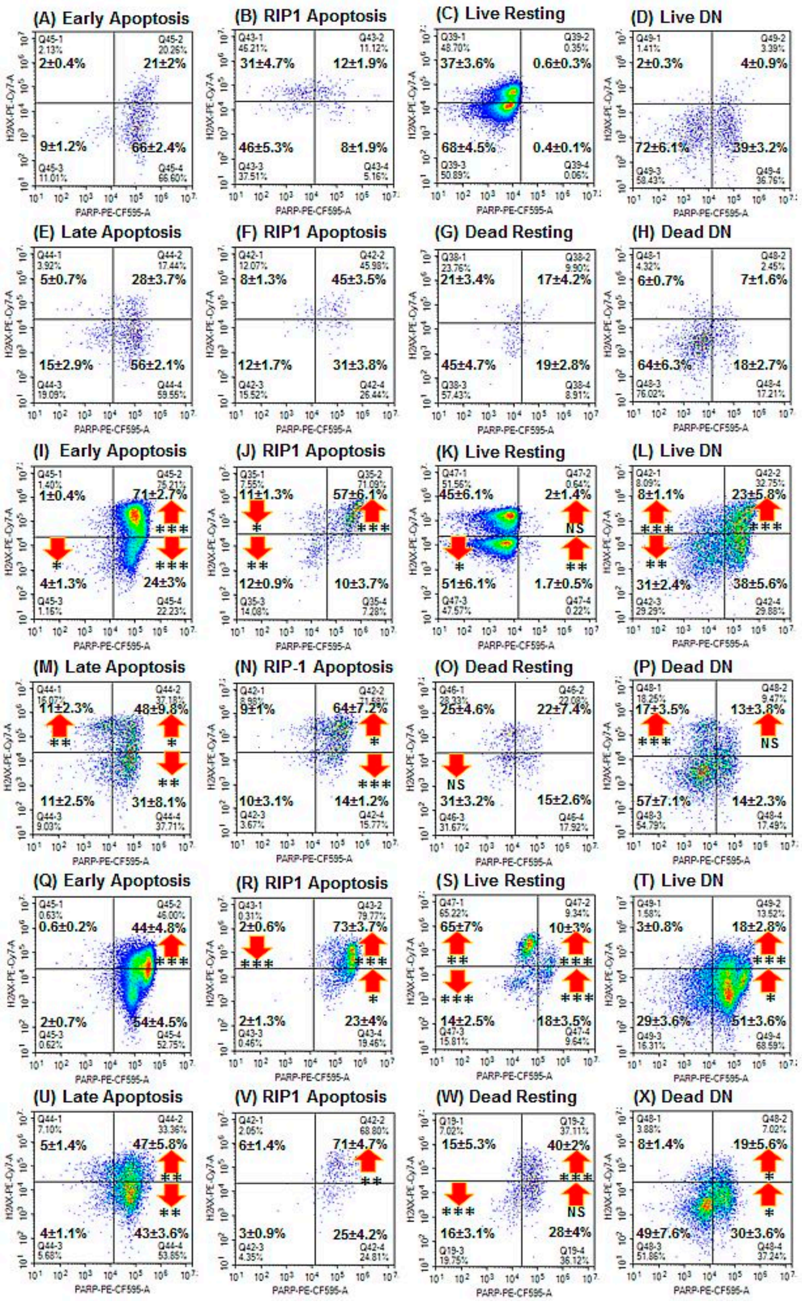

Figure 2. pH2AX hyper-activation of cleaved PARP/parthanatos, apoptosis via cleaved PARP and DNA Damage assay. Jurkat cells were incubated with live-fix dye, Zombie NIR, fixed and permeabilised and incubated with anti-active caspase-3-BV650, RIP3-PE, cleaved PARP-PE-CF595, pH2AX-PE-Cy7 according to the Materials and Methods and 100,000 cells analysed flow cytometrically. After gating on live and dead cells from a Zombie NIR vs Caspase-3-BV650 dot-plot untreated (A-H), 0.25 % NaN_3 (I-P) or 1 µM Etop (Q-X) treated live and dead Jurkat cells were analysed on a RIP3-PE vs Caspase-3-BV650 dot-plot. The early and late apoptosis phenotypes indicated by RIP3^{-ve}/Caspase-3^{+ve} (A, E, I, M, Q, U), RIP1-dependent apoptosis phenotype indicated by RIP3^{+ve}/Caspase-3^{+ve} (B, F, J N, R, V), resting phenotype indicated by RIP3^{+ve}/Caspase-3^{-ve} (C, G, K, O, S, W), or double negative indicated by RIP3^{-ve}/Caspase-3^{-ve} (D, H, L, P, T, X), respectively. Such live and dead populations were then analysed for cleaved PARP and pH2AX. n=3, % mean ± % SEM, student t test NS (not significant), *P*<0.05*, *P*<0.01**, *P*<0.001***, with arrows indicating change compared to untreated cells.

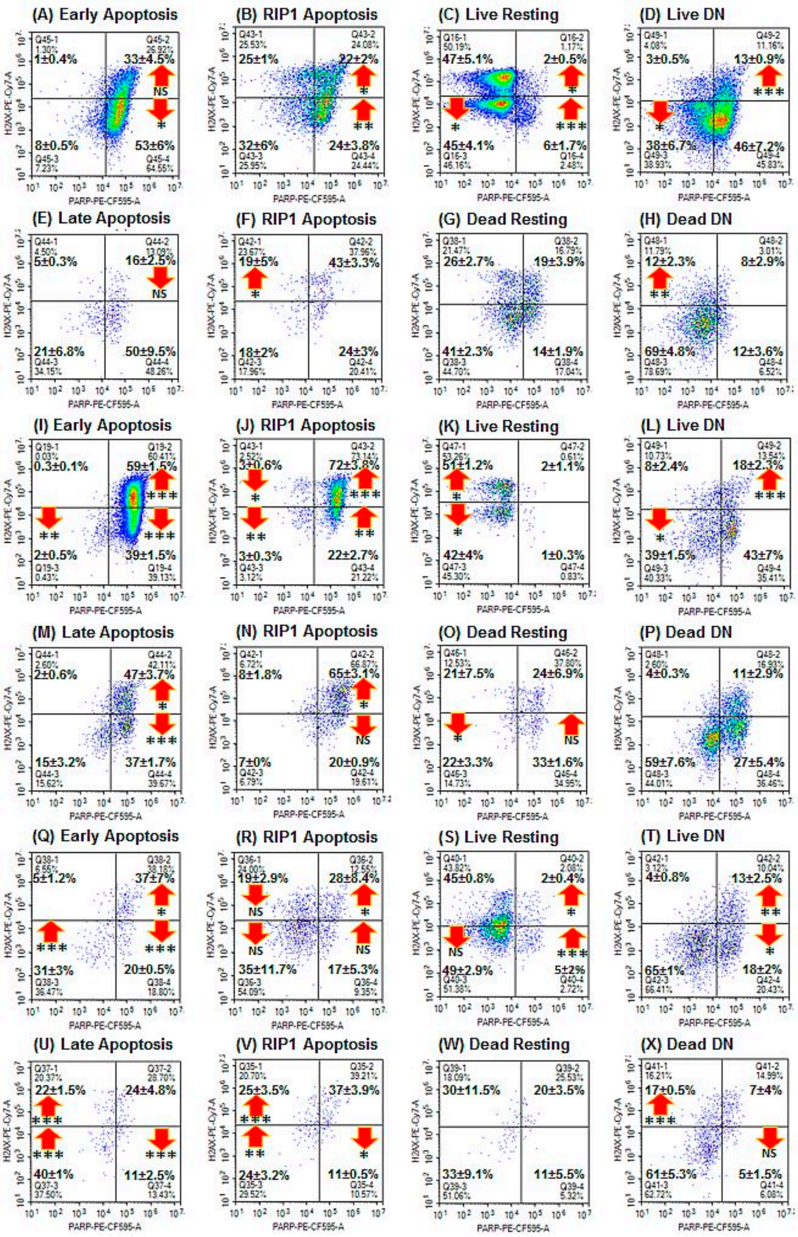

Figure 3. pH2AX hyper-activation of cleaved PARP/parthanatos, apoptosis via cleaved PARP and DNA Damage of blocked oncosis. Jurkat cells were pre-treated zVAD (20 µM) and or Nec-1 (60 µM) for 2 h then incubated with 0.25 % NaN_3 for 24 h. Cells were then loaded with live-fix dye, Zombie NIR, fixed and permeabilised and incubated with anti-active caspase-3-BV650, RIP3-PE, PARP-PE-CF595, and pH2AX-PE-Cy7, according to the Materials and Methods and 100,00 cells analysed flow cytometrically. After gating on live and dead cells from a Zombie NIR v Caspase-3-BV650 dot-plot treated live and dead Jurkat cells with zVAD with 0.25 % NaN_3 (A-H), Nec-1 with 0.25 % NaN_3 (I-P) or zVAD with Nec-1 and 0.25 % NaN_3 (Q-X) were analysed on a RIP3-PE vs. Caspase-3-BV650 dot-plot. The early and late apoptosis phenotype indicated by RIP3^{-ve}/Caspase-3^{+ve} (A, E, I, M, Q, U), RIP1-dependent apoptosis phenotype indicated by RIP3^{+ve}/Caspase-3^{+ve} (B, F, J N, R, V), resting phenotype indicated by RIP3^{+ve}/Caspase-3^{-ve} (C, G, K, O, S, W), or double negative indicated by RIP3^{-ve}/Caspase-3^{-ve} (D, H, L, P, T, X), respectively and such live and dead populations were then analysed for PARP and pH2AX. n=3, % mean ± % SEM, student t test NS (not significant), *P*<0.05*, *P*<0.01**, *P*<0.001***, with arrows indicating change compared to untreated cells.

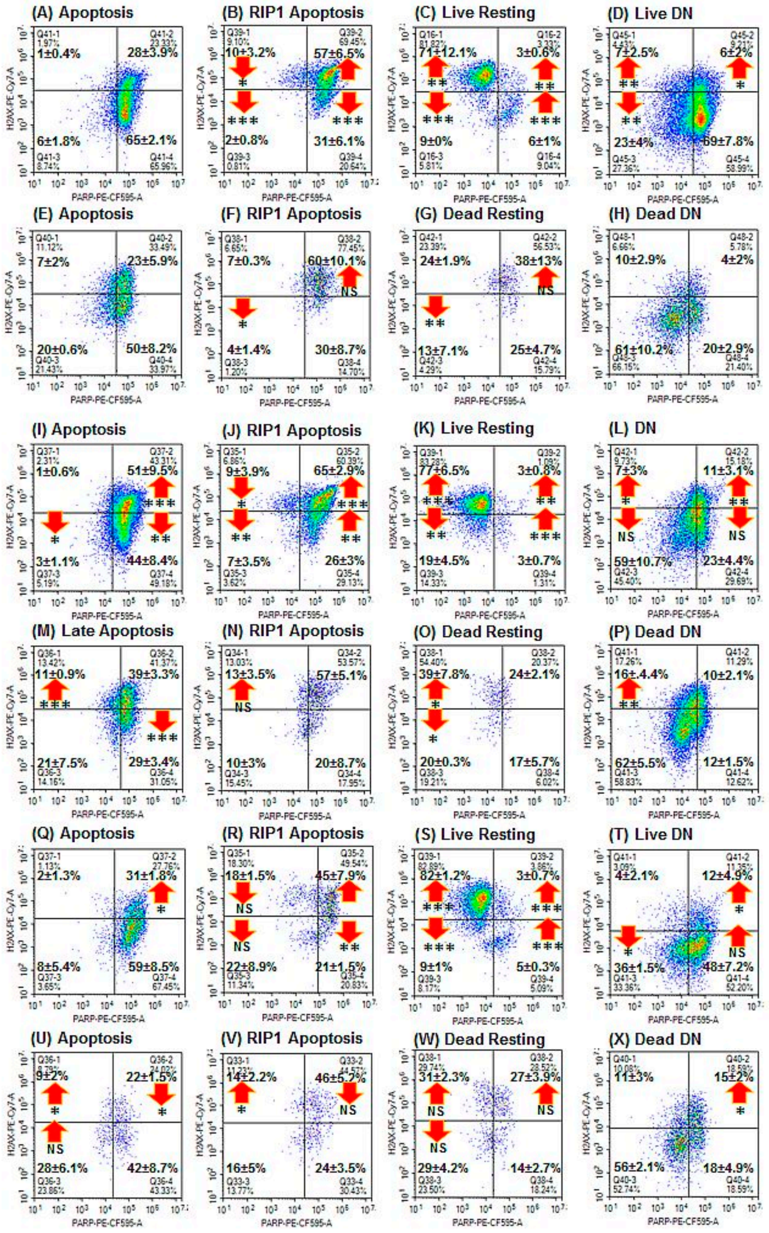

Figure 4. pH2AX Hyper-activation of cleaved PARP/parthanatos, apoptosis via cleaved PARP and DNA Damage blocked apoptosis. Jurkat cells were pre-treated zVAD (20 µM) and or Nec-1 (60 µM) for 2 h then incubated with 1 µM Etop for 24 h. Cells were then loaded with live-fix dye, Zombie NIR, fixed and permeabilised and incubated with anti-active caspase-3-BV650, RIP3-PE, PARP-PE-CF595, and pH2AX-PE-Cy7, according to the Materials and Methods and 100,00 cells analysed flow cytometrically. After gating on live and dead cells from a Zombie NIR v Caspase-3-BV650 dot-plot treated live and dead Jurkat cells with zVAD with 0.5 µM Etop (A-H), Nec-1 with 1 µM Etop (I-P) or zVAD with Nec-1 and 1 µM Etop (Q-X) were analysed on a RIP3-PE vs Caspase-3-BV650 dot-plot. The early and late apoptosis phenotype indicated by RIP3^{-ve}/Caspase-3^{+ve} (A, E, I, M, Q, U), RIP1-dependent apoptosis phenotype indicated by RIP3^{+ve}/Caspase-3^{+ve} (B, F, J N, R, V), resting phenotype indicated by RIP3^{+ve}/Caspase-3^{-ve} (C, G, K, O, S, W), or double negative indicated by RIP3^{-ve}/Caspase-3^{-ve} (D, H, L, P, T, X), respectively and such live and dead populations were then analysed for cleaved PARP and pH2AX. n=3, % mean ± % SEM, student t test NS (not significant), P<0.05*, P<0.01**, P<0.001***, with arrows indicating change compared to untreated cells.
